# Inpatient Rheumatology Consultation Prompted by Positive Autoantibodies in Patients Receiving Intravenous Immunoglobulin Therapy: A Case Series and Literature Review

**DOI:** 10.7759/cureus.37008

**Published:** 2023-04-01

**Authors:** Zarlasht Fnu, Asif Uddin, Brianne Navetta-Modrov, Asha Patnaik, Alan Kaell

**Affiliations:** 1 Medicine, Mather Hospital/Northwell Health, Port Jefferson, USA; 2 Medicine, Renaissance School of Medicine, Stony Brook University, Stony Brook, USA; 3 Medicine, Zucker School of Medicine/Northwell Health, Hempstead, USA

**Keywords:** anti-nuclear antibody (ana), immune thrombocytopenic purpura (itp), autoimmune hemolytic anemia (aiha), guillain-barre syndrome (gbs), intravenous immunoglobulin (ivig), autoantibodies

## Abstract

Intravenous immunoglobulin (IVIG) is a therapeutic preparation used in the treatment of multiple diseases. Autoimmune testing with antinuclear antibody (ANA) screening is often obtained for some of these conditions, but only after initiation of IVIG treatment. This can present a diagnostic dilemma in hospitalized patients and may trigger a rheumatology consultation. We describe our consultative inpatient two-year experience with five such patients and review the pertinent literature.

A retrospective chart review of rheumatology inpatient consultations between 6-2018 and 6-2020 at our academic tertiary care hospital for post-IVIG positive serologies was performed. A pertinent literature review was performed. Five patients had a positive ANA and other autoantibodies detected in their serum after they received IVIG for non-rheumatological conditions. None of these patients met the criteria for a connective tissue disease. The literature review identified a total of 58 patients from case reports and case series, several of whom tested positive for ANA and other antibodies after receiving IVIG. Studies assessing specific IVIG products detected multiple autoantibodies in the donor pool.

Autoimmune testing is initiated on inpatients receiving IVIG for non-rheumatological conditions. If an autoantibody ANA screen is positive, a rheumatology consultation may be requested. In the absence of pre-IVIG antibody tests it is difficult to interpret post-IVIG-positive antibodies. Whether such positive antibodies are of clinicopathological significance is determined by clinical judgment and time.

## Introduction

Intravenous immunoglobulin (IVIG) is a blood product prepared from multiple human plasma donations, consisting of unmodified immunoglobulin G antibodies that modulate the immune system of the recipient [1]. Therapeutic benefits for multiple clinical conditions including immune thrombocytopenia (ITP), primary immunodeficiency diseases, and dermatomyositis exist [2]. Autoimmune testing with antinuclear antibody (ANA) screening commonly obtained for patients presenting with isolated conditions warranting empiric IVIG is sometimes drawn after initiation of treatment. If positive autoantibodies are found post-IVIG in these patients, this can prompt rheumatology consultation. Positive autoantibodies in such settings may be determined to be falsely positive if a thorough history, physical examination, and laboratory evaluation exclude a rheumatological disorder.

The Choosing Wisely initiative of the American Board of Internal Medicine (ABIM) in conjunction with the American College of Rheumatology (ACR) listed five practices in rheumatology that should be questioned to improve value-based care [3]. Avoid testing ANA sub-serologies if ANA is negative and clinical suspicion of immune-mediated disease is low. The American Society for Clinical Pathology and the American Society for Clinical Laboratory Science have similar recommendations [4].

Little is known about the development of false-positive autoantibodies secondary to IVIG infusion in non-rheumatological conditions. Herein we report a case series of five patients who had autoantibodies detected after having received IVIG without prior measurement of such antibodies. We also provide the pertinent literature review regarding this clinical challenge.

## Case presentation

A retrospective chart review of patients who were hospitalized between June 2018 and June 2020 was performed. The patients were selected after meeting these three criteria: 1) their primary team requested an adult rheumatology consultation when an ANA serology was positive; 2) no patients had a recognized pre-existing rheumatologic disease; and 3) they previously received or are currently receiving IVIG therapy for a non-rheumatologic condition.

We searched the databases of PubMed (MEDLINE, National Library of Medicine, and PubMed Central), Web of Science, and Google Scholar from inception to October 2022 using the terms “intravenous immunoglobulin,” "autoantibodies,” and “antinuclear antibodies (ANA).”

Five patients met our inclusion criteria (see Table 1). Ages ranged from 35 to 75 years with three females and two males. Admitting diagnoses included Guillain-Barre syndrome, ITP, and autoimmune hemolytic anemia. All of them had autoimmune testing after initiating treatment with IVIG. Patient 1 received four doses of IVIG. Patient 2 was receiving IVIG every two weeks for ITP. Patients 3 and 4 received multiple doses of IVIG. Patient 5 received three doses of IVIG. All five tested positive for ANA at varying titers as well as anti-SS-A. Four out of five patients had abnormal complement levels. Patient 2 also tested positive for anti-ribonucleoprotein (RNP), double-stranded DNA (dsDNA), anti-Scl-70, and anti-RNA polymerase III antibodies. Patient 3 tested positive for an anti-cyclic citrullinated peptide (CCP) antibody. Patient 1 had repeat testing one month later which revealed a negative ANA and anti-SS-A.

**Table 1:** Lab results of patients who met the inclusion criteria. ANA: anti-nuclear antibody; SM: Smith; RNP: ribonucleoprotein; dsDNA: double-stranded DNA; RF: rheumatoid factor; CCP: cyclic citrullinated peptide.

Variables (units)	Patient 1	Patient 2	Patient 3	Patient 4	Patient 5	Upper limit of normal/Range
ANA, titer	1:40	1:640	1:320	1:320	1:80	<1:320
SS-A, units	32	38	25	25	52	<20
SS-B, units	4	10	8	7	12	<20
Anti-Sm, units/mL	5	20	5	14	9	<20
Anti-RNP, units	7	24	9	17	13	<20
Anti-dsDNA, IU/mL	14	38	9	-	24	<25
Anti-Scl-70, units	-	141	-	-	-	0-40
Anti-RNA polymerase III, units	29	-	-	-	-	<19
RF, IU/mL	-	10	13	<10	<11	<30
Anti-CCP, units	-	-	232	-	18	<20 U
Complement C3, mg/dL	116	-	42	113	49	76-164
Complement C4, mg/dL	10	-	2	15	3	16-49
ANCA, titer	1:20	-	-	-	-	<1:20

Our literature search yielded 142 results, but we included only those reports that involved patients receiving IVIG and being tested for autoantibodies including ANA. We identified one case report and two case series comprising a total of 58 patients. One study followed six patients with common variable immunodeficiency who received IVIG treatments and two out of six tested positive for ANA, with anti-SS-A antibodies positive in three, equivocal in two, and negative in one out of the six [5]. Ten mini-pools of IVIG preparations consisting of 480 donors in each pool were also tested for anti-SS-A antibodies with four out of 10 detecting anti-SS-A positivity. This prompted the testing of 40 mini-pools and found that the frequency of positive anti-SS-A antibodies in asymptomatic donors was 0.69%. In another study, 50 patients with various neurological conditions such as autoimmune encephalitis and myasthenia gravis receiving IVIG were tested for ANA and anti-neuronal antibodies [6]. Overall, 45 out of 50 (90%) of these patients had a positive ANA and anti-SS-A antibodies. Another report was on two patients (one with rheumatoid arthritis and one with immunodeficiency) receiving IVIG who tested positive for antibodies associated with hepatitis B and syphilis [7]. They also detected positivity of multiple infectious (e.g., hepatitis B surface and core antibody) and autoimmune (e.g., ANA, anti-dsDNA, and antineutrophil cytoplasmic antibody) serological tests after testing multiple IVIG products.

## Discussion

In this case series, we identified five inpatients with non-rheumatological conditions who tested positive for ANA and SS-A only after empiric IVIG administration. None of these patients had any known positive autoantibodies before receiving IVIG. Our inpatient consultative experience over this two-year period determined that none of the patients met the classification or diagnostic criteria for a connective tissue disease. One patient tested negative one month later. Three patients were deceased, and the fifth patient was unable to be located. Our literature review revealed multiple cases of patients testing positive for autoantibodies after receiving IVIG for non-rheumatological disorders [5,6]. We also found reports of various IVIG product testing that yielded similar antibodies detected from the donors [5].

It is unclear if the presence of these autoantibodies in recipients can lead to clinical symptoms of connective tissue diseases. ANA and SS-A antibodies have been detected in otherwise healthy people in the general population with low titers [8,9]. The role of the pathogenicity of these antibodies in the recipients’ system is unknown and warrants further research [10].

It is important to try differentiating false-positive and true-positive antibodies in the setting of recent IVIG therapy, with the true/false dichotomy concept related to clinical diagnosis at the point of care. This is accomplished through a detailed history, physical examination, and repeating the positive antibody laboratory tests several weeks after completion of IVIG therapy to assess the persistence or resolution of such antibodies and clinical evolution [11].

An intentional pragmatic, retrospective design limitation of our study was the unavailability of pre-IVIG autoantibodies. Such knowledge may provide clarity on the positive autoantibody tests that were detected only after the initiation of therapy. However, such real-world clinical situations occur in patients hospitalized acutely for treatment with empiric IVIG when first presenting with non-rheumatological disorders that benefit from IVIG but may present potential associated rheumatic illness. We were only able to obtain repeat autoantibody testing in one out of the five patients, which represents another potential limitation of our study but reflects the reality and challenges of continuity in care and follow-up.

## Conclusions

It is imperative that clinicians are aware of the diagnostic dilemma that can present if positive autoantibodies are detected after initiating IVIG. Such awareness in the spirit of the Choosing Wisely ABIM Campaign may diminish unwarranted diagnostic testing and improve the quality of care.
